# Post-Psychotic Depression: A Comprehensive Narrative Review

**DOI:** 10.3390/diseases14040150

**Published:** 2026-04-20

**Authors:** Karol Piotr Mirkowski, Kalina Aleksandra Hac, Zuzanna Kryś, Jerzy Leszek

**Affiliations:** 1 Student Scientific Association, Wroclaw Medical University, 50-367 Wroclaw, Poland; kalina.hac@icloud.com (K.A.H.); zuzanna.krys@gmail.com (Z.K.); 2 Department of Psychiatry, Wroclaw Medical University, 50-367 Wroclaw, Poland; jerzy.leszek@umw.edu.pl

**Keywords:** post-psychotic depression, schizophrenia, antipsychotics, insight, suicidality

## Abstract

Background: Post-psychotic depression (PPD) is an underestimated but clinically significant affective syndrome that occurs during remission from psychosis, particularly in schizophrenia. Material and Methods: This comprehensive review traces the evolution of this concept over five decades of research, starting from its initial differentiation from primary depression and schizoaffective disorders in the 1970s. Relying on more than thirty studies, we analyze historical definitions, biological and psychosocial mechanisms, diagnostic controversies, and therapeutic implications. Results: Research indicates that PPD develops from multiple contributing factors, including psychological insight, autobiographical disturbances, pharmacological influences, and social losses, rather than simply as a byproduct of psychosis or pharmacological treatment. We discuss the persistence of depressive symptoms after acute remission, their role in suicidal tendencies, and the diagnostic challenges arising from the overlap of negative symptoms and demoralization. Despite its exclusion from current diagnostic standards, PPD continues to affect a significant fraction of patients, particularly those with high insight and early onset. Conclusions: Effective treatment requires a multidimensional, phase-specific approach combining antidepressants, atypical antipsychotics such as lurasidone, and psychological interventions targeting identity, self-esteem, and narrative processing. We argue that PPD should be reintroduced as a distinct clinical unit and incorporated into psychiatric guidelines to reduce diagnostic oversights and improve patient outcomes.

## 1. Introduction

Despite the fact that Mayer-Gross was the first to conceptualize PPD, describing it as despair or denial of the future in response to a psychotic episode, the recognition of PPD as a distinct clinical entity can be traced back to the seminal work of McGlashan and Carpenter. They described PPD as a phase that often follows the remission of acute psychotic symptoms in schizophrenia, highlighting characteristic features such as withdrawal, muteness, and marked loss of motivation. Importantly, they estimated that approximately one-quarter of patients recovering from a psychotic episode developed this syndrome, which they argued was distinct from both schizoaffective disorder and major depressive disorder. It also appeared more likely in ‘reactive’ schizophrenic patients with no prior history of illness [1].

The nosological status of PPD remains unclear. Guerrero-Jiménez et al. claimed that although the concept is recognized in clinical practice, it has never been formally included as a separate diagnostic entity in major classification systems. In the DSM-IV, PPD was not recognized as an official diagnosis. Instead, it was listed in Appendix B as a condition requiring further study. Patients with symptoms of depression following psychosis were typically diagnosed with schizophrenia and unspecified depressive disorder. Importantly, PPD was not included in the DSM-5, reflecting the continuing uncertainty regarding its diagnostic validity [2].

More broadly, recent changes in psychiatric classification systems, including ICD-10 and ICD-11, reflect a shift toward a dimensional approach that emphasizes symptom domains rather than distinct diagnostic entities. Within this framework, depressive symptoms occurring after psychosis are typically viewed as part of a broader spectrum of psychotic and affective disorders, rather than as a distinct condition.

This lack of clarity complicates both clinical practice and research, as PPD remains difficult to distinguish from depressive symptoms occurring in the course of schizophrenia.

Over subsequent decades, researchers expanded upon this concept, exploring biological, psychological, and social determinants.

## 2. Methods

This literature review is based on a comprehensive search of the PubMed database covering the period from 1976 to 2025. Approximately 35 key studies were included in the final analysis. Only articles published in English were included. Articles without full text, publications in languages other than English, and conference abstracts were excluded. The search included keywords such as post-psychotic depression, schizophrenia, psychosis, depressive symptoms, and treatment with antipsychotic medications.

The initial search provided a wide range of publications. After analyzing titles and abstracts for relevance and then screening the full texts, studies directly addressing the conceptualization, mechanisms, and clinical aspects of PPD were selected for the review. Reference lists of the included articles were also reviewed to identify additional relevant studies.

This review was designed as a narrative summary rather than a systematic review. Given the conceptual heterogeneity of PPD, the historical evolution of this construct, and the lack of a standard operational definition, a narrative approach was considered more appropriate for integrating clinical, psychological, neurobiological, and phenomenological perspectives. No formal quality assessment, pre-registration, or quantitative synthesis was conducted.

As a narrative review, this study is subject to selection bias. However, efforts were made to include representative and significant studies from different time periods and based on various conceptual frameworks, in order to provide a balanced overview of this field.

## 3. Results

### 3.1. Historical Perspectives on Post-Psychotic Depression (1976–1992)

Subsequent work by Das and Kapur reinforced the concept of PPD through a prospective study in India. They conceptualized PPD as a largely reactive condition emerging in response to the psychological consequences of psychosis, while also acknowledging potential depressogenic effects of early antipsychotics—particularly phenothiazines. Their findings emphasized the heterogeneity of PPD and the need for operational definitions to differentiate it from primary mood disorders [3].

By the late 1980s, research had begun to expand into Western cohorts. Berrios and Bulbena examined the Fulbourn cohort in the UK and identified specific differences between patients with and without PPD. They noted that those who developed PPD tended to be older at onset and had a higher prevalence of auditory hallucinations. The authors reported that patients with PPD were readmitted to the hospital more frequently, though their length of admission was shorter than that of controls. Importantly, they found no evidence that depot neuroleptics increased the risk of PPD, challenging earlier assumptions that medication played a central role in its etiology [4].

At the same time, attention turned toward the longitudinal course of depressive symptoms in schizophrenia. Leff, Tress, and Edwards observed that depressive states could appear not only after the resolution of psychosis but also during its course. They emphasized the variability of depressive trajectories and underscored the diagnostic challenges posed by the overlap between negative symptoms, medication side effects, and genuine depression. Importantly, the authors found no evidence that haloperidol exacerbated depressive symptoms in most patients with schizophrenia, with some evidence suggesting symptom attenuation [5]. Becker advanced this debate by presenting secondary depression in schizophrenia as a potentially distinct syndrome rather than a milder form of major depressive disorder. In his work, he emphasized the predominance of cognitive features, for example, hopelessness, helplessness, guilt, suicidal ideation, as more specific markers of secondary depression in schizophrenia than somatic features such as insomnia or retardation, and questioned whether the existing diagnostic criteria capture these presentations [6].

It was also a productive time in Japan, where Mino and Ushijima introduced the concept of “post-psychotic collapse,” a closely related phenomenon characterized by profound inactivity, loss of vitality, and demoralization following the resolution of acute psychosis. They reported a strikingly high frequency of this syndrome observed in more than 80% of their cohort. The authors discovered that the duration of PPC was strictly associated with the length of the acute psychotic phase. Longer hospitalization and environmental influences could increase demoralization by contributing to loss of employment and social position, which in turn may exacerbate depressive states and inactivity [7]. This work broadened the scope of inquiry by linking social context and institutionalization to the onset and persistence of PPD-like states.

Siris extended the investigation to biological markers, examining the role of thyroid-releasing hormone (TRH) tests in patients with PPD. Although his findings did not establish definitive biological correlates, this research reflected the growing interest in identifying physiological dimensions of PPD alongside psychosocial explanations [8].

During the early 1990s, methodological refinements improved the reliability of studying depressive symptoms in schizophrenia. Addington et al. developed and validated a depression rating scale, “The Calgary Depression Scale”, specifically for use with schizophrenic patients, helping to reduce the long-standing problem of conflating negative symptoms with depressive states [9].

Around the same time, Chintalapudi, Kulhara, and Avasthi conducted a clinical study in India that provided further evidence of the psychosocial underpinnings of PPD. They reported that patients with PPD had experienced a greater number of stressful life events and reported lower perceived social support than non-PPD patients and greater somatization, anxiety, and sadness during acute psychosis. The authors also highlighted the role of family structures, especially the big impact of nuclear families [10].

The division of research on PPD into historical periods was based more on significant conceptual and clinical changes documented in the literature than on strict chronological boundaries. Early studies focused on descriptive and phenomenological aspects of PPD, while later research increasingly emphasized cognitive, neurobiological, and integrative models.

It is also possible that changes in publication frequency over time may be partly related to changes in diagnostic frameworks, including the absence of PPD as a formal diagnostic entity in the DSM classifications. The lack of formal recognition may have contributed to the reduced visibility of this concept in subsequent literature, although this relationship remains speculative.

### 3.2. Mechanisms and Risk Factors (1993–2006)

As the concept of PPD matured in the 1990s, studies questioned whether PPD is primarily a psychological response to psychosis, a neurobiological consequence of treatment, or a combination of these and other factors.

An important step in the conceptualization of PPD was its incorporation into a cognitive model of assessment and self-assessment by Birchwood et al. To clarify temporal relationships, the authors defined the “pre-PPD” state as the last observation period before the onset of depressive symptoms. This ensured that the observed cases of depression were new episodes rather than continuations of earlier phases [11]. The authors argued that depression following psychosis often results from the way patients interpret their illness. The assessment of loss, humiliation, and imprisonment, combined with heightened feelings of guilt, was strongly associated with subsequent episodes of depression. These processes exacerbated low self-esteem, a sense of “lowered status” in the social hierarchy, and increased self-criticism, which distinguished PPD from depressive states observed in other psychiatric contexts [12].

A year later, Harrison et al. added a complementary perspective through long-term observation of psychotic illnesses. They emphasized the interaction between recovery and social functioning, noting that poorer psychosocial outcomes were associated with a higher risk of depressive states after psychosis, while also drawing attention to neurodevelopmental factors [13]. These findings confirmed earlier observations that prolonged hospitalization and loss of social roles contributed to demoralization [7].

Clinical reviews at the turn of the century broadened the discussion on biological and pharmacological mechanisms. Siris et al. emphasized the importance of recognizing depression in schizophrenia as a treatable condition, while acknowledging the difficulties in distinguishing it from negative symptoms and medication side effects [14]. Jeczmien et al. also highlighted the heterogeneity of PPD symptoms, suggesting that both affective vulnerability and psychotic processes contribute to its development. Controlled studies suggest that antidepressants may be effective in treating PPD in both the short- and long-term, although their effect is not as strong as in primary depressive disorders. Importantly, discontinuation of antidepressants often led to a recurrence of both depression and psychosis [15].

Further empirical studies conducted in the early 21st century examined specific biological and clinical correlates. Bressan et al. documented the occurrence of depressive episodes in stable schizophrenia, highlighting their frequency even beyond the acute period after remission. The study shows that depression in schizophrenia is not limited to the first year after psychosis, challenging the ICD-10 criteria, which limit the diagnosis to 12 months. The authors argue that limiting depression to “post-psychotic” phases leads to misclassification and prevents patients from receiving appropriate treatment, both pharmacological and psychotherapeutic [16]. Similarly, Candido and Romney compared the symptoms of depression in paranoid and non-paranoid schizophrenia with those observed in major depressive disorders, concluding that secondary depression in schizophrenia is a separate syndrome and not a variant of primary depression [17]. Kohler and Lallart presented a historical review that consolidated these perspectives, highlighting how the recognition of PPD has evolved over decades and identifying it as an important area of both scientific research and clinical care [18].

The role of autobiographical memory and self-perception has been examined in more detail by Iqbal et al. In their study of first-episode psychosis, they showed that patients who developed PPD often recalled their past in a more negative way and had difficulty constructing positive narratives about themselves [19]. These findings complemented Birchwood’s earlier model [11,12], supporting the view that cognitive and self-referential processes play a key role in the onset of PPD. Birchwood et al. later elaborated on these mechanisms, proposing that depression in schizophrenia is best understood through psychological pathways involving self-esteem, loss of social standing, and illness awareness [20].

During this period, research also began to focus on adolescence and the first episode of psychosis as key moments in the development of PPD. Schwartz-Stav et al. suggest revisiting the DSM-IV criteria, proposing less focus on somatic and behavioral symptoms and more on cognitive and affective criteria when diagnosing PPD in adolescents. The authors studied adolescents with schizophrenia and found that depression and suicidal thoughts often occurred after the acute psychosis had subsided [21]. Similarly, Oosthuizen et al. found that symptoms of depression were closely related to remission outcomes in first-episode patients [22].

**Figure 1:**
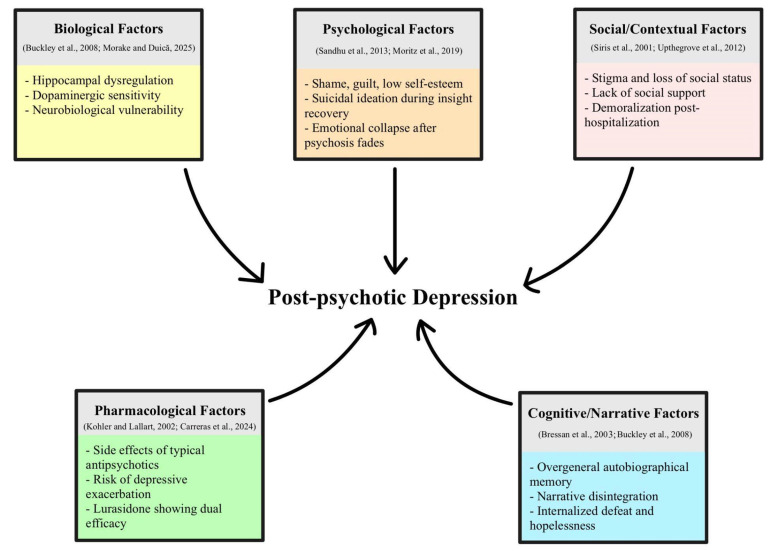
Key domains contributing to PPD. A schematic representation of five major interacting mechanisms underlying PPD: biological factors (hippocampal dysregulation, dopaminergic sensitivity, neurobiological vulnerability), psychological factors (shame, guilt, low self-esteem, suicidal ideation emerging with insight), social/contextual factors (stigma, loss of social status, poor support, post-hospitalization demoralization), pharmacological factors (side effects of typical antipsychotics, risk of depressive exacerbation, potential dual efficacy of lurasidone), cognitive/narrative factors (overgeneral autobiographical memory, narrative disintegration, internalized defeat) [14,16,18,23,24,25,26,27,28].

In summary, studies conducted between 1993 and 2006 showed that PPD cannot be explained by a single mechanism. Psychological models emphasized the role of loss, humiliation, and self-blame [11,12,19,20], while clinical and long-term studies pointed to the influence of social context, recovery process, and adolescence as key risk factors [13,21,22]. Biological studies confirmed the possible influence of pharmacological factors, but failed to establish a simple drug-induced model [14,16,17,18]. Over the past decades, PPD began to be viewed as a multidimensional syndrome, summarized in Figure 1, which combines five major areas related to the development of PPD—biological, psychological, social/contextual, pharmacological, and cognitive/narrative factors—highlighting how these mechanisms interact to cause depressive states during remission.

### 3.3. Contemporary Research and Clinical Practice (2007–2024)

In the years following 2006, research on PPD increasingly reflected a broader shift in psychiatric approaches toward integrated, biopsychosocial models of illness. Researchers began to analyze not only the prevalence and symptomatology of PPD, but also its interaction with insight, narratives of recovery, and the course of the first episode of psychosis.

Conley et al. demonstrated that depressive symptoms remain a persistent burden in schizophrenia, affecting approximately one-third of patients over a three-year follow-up period. These symptoms were associated with impaired functioning, reduced adherence to treatment, and a higher risk of relapse, occurring independently of positive symptom control and changes in pharmacological treatment [29]. Buckley et al. further emphasized that up to half of patients with schizophrenia experience symptoms of depression, especially in the post-psychotic phase. The authors discussed how treatment with both typical and atypical antipsychotic drugs may contribute to or exacerbate symptoms of depression, complicating diagnosis and treatment. They also pointed to neurobiological research linking changes in the hippocampus to depressive states, suggesting a partial overlap with affective disorders [23].

The clinical significance of these findings is supported by reports of cases of serious self-harm occurring during remission. A case described by Bhattacharyya et al., in which a patient with paranoid schizophrenia committed serious self-mutilation of the genitals during remission, illustrated the risk of overlooking severe depressive states in the absence of psychosis, and drew attention to the increased risk of self-harm during recovery [30].

At the same time, scientific research has begun to place increasing emphasis on the psychological and social aspects of health recovery. Jackson et al. examined the psychological consequences of recovery and identified shame and internalized stigma as key factors in the development of depressive reactions. Their study, focusing on the early stages of recovery, found that many patients perceived their psychotic experiences as traumatic, resulting in post-traumatic reactions and persistent affective distress [31].

Upthegrove et al. further explored how sociocultural interpretations of psychosis influence outcomes of depression treatment. He found that both patients and caregivers constructed narratives based on failure, loss, and stigma that correlated with the severity of PPD. Surprisingly, some ethnic groups had lower rates of depression despite worse clinical outcomes, suggesting that cultural narratives may soften affective breakdown without changing the course of the illness [24].

These findings were further confirmed by qualitative research. In Sandhu et al.’s qualitative study, photo-elicitation interviews were used to explore subjective experiences of remission. Participants described their post-psychotic state not in biological or somatic terms, but as a loss of meaning in existence, marked by shame, confusion, and fear of relapse. Such relationships highlighted the limitations of conventional diagnostic criteria and the need to consider how PPD manifests itself through identity breakdown rather than just through the return of symptoms [25].

A psychoanalytic explanation of PPD was presented by Potik, who proposed that in paranoid schizophrenia, delusions serve as a narcissistic defense. When psychosis subsides and defense mechanisms disappear, patients experience intense sensitivity, helplessness, and existential anxiety. This exposure often leads to depression, not as a set of symptoms, but as a reaction to the loss of mental structure and autonomy [32].

Subsequent studies have focused on therapeutic and diagnostic implications. Gregory and Mallikarjun performed a systematic review of the efficacy of antidepressants in schizophrenia, focusing on PPDs. Their results showed that selective serotonin reuptake inhibitors, particularly citalopram and sertraline, had some benefits in treating depressive states following psychosis, but the data remained inconsistent. Importantly, they found no evidence that antidepressant treatment exacerbates psychotic symptoms, although the boundaries between PPD, primary depression, and negative symptoms remained problematic from a diagnostic standpoint [33].

The clinical framework was also analyzed in a comparative study by Dondé et al., who examined national guidelines from several countries. Although most guidelines recognized the high prevalence of depressive symptoms in schizophrenia, very few specifically addressed PPD. Guidelines often do not reflect the perspective of patients, even though patients consistently prioritize the relief of depression more than clinicians do. The inclusion of patients as stakeholders in the guideline development process was identified as an urgent need [34].

Moritz et al. proposed a cognitive framework in which symptoms of depression often occur when patients begin to process the social and emotional impact of the disease. According to this model, paranoid delusions can work as a psychological buffer against self-awareness. When paranoia subsides, patients are forced to confront personal failures, relationship breakdowns, and stigmatizing experiences, which in turn can trigger depressive episodes during remission [26]. Similar to earlier psychoanalytic observations by Potik, the breakdown of psychosis represents a critical transition point in psychological development, through the loss of protective illusions or a painful return to self-awareness, ultimately exposing the individual to depressive affect [32].

Despite the removal of PPD from DSM-5, Guerrero-Jiménez et al. argued that PPD remains a clinically important and significant concept. In his clinical review, he noted that the prevalence of the disorder ranged from 25% to 44% in the general population of individuals with schizophrenia and up to 50% in individuals experiencing a first episode of psychosis. Risk factors included high levels of insight, stressful life events, poor social support, and low premorbid functioning. The author maintained that PPD has important prognostic value and that abandoning the term risks missing an important clinical reality. His review mentioned that the Calgary Depression Scale has been found to be the most reliable tool for distinguishing PPD from negative symptoms or antipsychotic medication side effects [2].

Some pharmacological interventions have shown promise in individual cases. Oguchi et al. described a patient with severe, treatment-resistant PPD who did not respond to several antidepressants and atypical antipsychotics [35]. Switching to monotherapy with lurasidone resulted in complete remission of depressive symptoms and improvement in overall functioning, while Carreras et al. confirmed the validity of this approach in a prospective study of patients recovering from substance-induced psychosis. Symptoms of depression consistent with PPD appeared in 38% of subjects within six months of remission, but only 10% of those treated with lurasidone experienced depressive complications [27]. These cases indicate that antipsychotic drugs with antidepressant properties may play a role in future therapeutic strategies for PPD.

A recent neurobiological case report published by Morake and Duică is changing the perception of PPD as a purely reactive phenomenon, pointing to permanent changes in prefrontal-limbic circuits, HPA axis disorders, and persistent glutamatergic and dopaminergic imbalances after the symptoms have subsided. A clinical case study emphasizes that it may be necessary to target both circuits and psychosocial repair [28].

In summary, studies conducted between 2007 and 2025 confirm that PPD is a phenomenon resulting from a combination of biological vulnerability, psychological trauma, and sociocultural interpretation. Although PPD no longer has a formal diagnostic status, its clinical importance remains clear. Effective treatment and prevention require not only pharmacological measures, but also sensitivity to the meaning, memory, and personal consequences of remission.

### 3.4. Suicidality in Post-Psychotic Depression

One of the most clinically significant consequences of PPD is suicidality, which is consistently reported across different groups and clinical contexts.

Birchwood et al. were among the first to describe how the emergence of awareness of one’s own diagnosis, particularly after the remission of psychosis, can lead to suicidal thoughts and behaviors. They suggested that although greater awareness may predict better adherence to therapeutic recommendations, it also exposes patients to the full existential burden of their illness, often causing depression, hopelessness, and in some cases suicidal thoughts [11,12]. This insight paradox has become a recurring theme in research on suicidal tendencies after psychosis.

The relationship between suicidal tendencies and insight was also examined in detail by Schwartz-Stav et al., who studied adolescents in remission from schizophrenia and found that suicidal tendencies were closely related to the level of post-psychotic insight. Patients who regained insight into their situation were more likely to report suicidal thoughts, especially when accompanied by symptoms of depression and cognitive reinterpretation of losses associated with the illness [21]. These findings confirmed earlier claims that suicidal tendencies are not simply an extension of psychosis or severe depression, but are the result of their interaction, often manifesting during remission when mental resilience is reduced.

Meanwhile, cognitive approaches have led to a better understanding of the mechanisms underlying vulnerability to risks. Iqbal et al. contributed to this discussion by focusing on the autobiographical memory patterns of patients after their first episode of psychosis. Their research showed that individuals who developed PPD and suicidal thoughts tended to have more segmented and negative memories of the past, with limited access to specific positive memories and greater overgeneral autobiographical memory (OGM). This memory style, already linked to suicidal tendencies in mood disorders, has been proposed as a cognitive vulnerability factor in schizophrenia as well. The authors hypothesized that impaired narrative coherence might impede coping, particularly in individuals who have lost social status or experience isolation [19].

The clinical relevance of these mechanisms is illustrated by case-based and qualitative studies. The clinical case described by Bhattacharyya et al. demonstrates that suicidal tendencies may be strongest not during psychosis itself, but after its remission, when the affective breakdown is no longer masked by delusions. Jackson et al. also argued for understanding suicidal tendencies after psychosis in the context of trauma. Based on qualitative interviews, he found that the recovery phase is often associated with a delayed confrontation with what patients perceived as a terrifying and humiliating experience. For some, the psychosis itself was an experience of being attacked, and suicide became a way to escape not from the voices or paranoia, but from the memory of that experience and the shame that followed [30,31].

These findings are further supported by sociocultural factors. Upthegrove et al. provided further evidence that suicidal tendencies in PPD result not only from emotional distress, but also from the meaning attached to mental breakdown. In his study, the strongest predictors of suicidal ideation were feelings of failure, entrapment, and hopelessness about the future, especially among patients with high levels of self-awareness and severe symptoms of depression. Importantly, suicidal tendencies rarely occurred in isolation. They were grounded in a broader framework of psychological interpretation, social context, and emotional loss [24].

Collectively, these findings suggest that suicidal tendencies in PPD are best understood not as an isolated symptom, but as the culmination of multiple converging processes: painful insight recovery, collapse of compensatory delusions, loss of social identity, and internalization of stigma. Psychological models, especially those emphasizing shame, failure, and autobiographical disintegration, seem particularly relevant in capturing the details of suicidal risk in this phase. From a clinical perspective, this highlights the need for targeted assessment and intervention in the early stages of remission, when affective sensitivity is often at its highest and protective structures are no longer functioning.

### 3.5. Differential Diagnosis of Post-Psychotic Depression

Accurate identification of PPD remains a persistent diagnostic challenge, mainly due to the overlap of its symptoms with other affective, psychotic, and iatrogenic disorders.

Becker et al. argued that secondary depression in schizophrenia, including PPD, should not be understood only as a milder form of major depressive disorder. Instead, he proposed that depressive symptoms such as hopelessness, guilt, and suicidal thoughts should dominate the clinical picture more than classic somatic symptoms such as insomnia or psychomotor retardation. According to Becker, this set of symptoms reflects a distinct affective process that requires separate diagnostic attention. Following these early attempts to better define depressive symptoms in schizophrenia, more precise diagnostic tools were developed. A few years later, Addington et al. contributed to developing the Calgary Depression Scale, which was specifically designed to distinguish symptoms of depression in schizophrenia from negative symptoms and medication side effects. The scale excluded somatic and cognitive items that were difficult to distinguish from psychotic features, improving diagnostic clarity and inter-individual reliability [6,9]. Its introduction remains a key methodological advance in differential diagnosis research.

Further research expanded on these findings by comparing depressive symptoms across different clinical presentations. Candido and Romney compared patients with paranoid and non-paranoid schizophrenia to those with major depressive disorder. They found that depressive symptoms—particularly cognitive and affective—were more pronounced in paranoid schizophrenia than in non-paranoid types, and in some areas, closely resembled those seen in major depression. Their results suggest that depressive features in schizophrenia may require distinct clinical attention, especially in the post-psychotic phase, where they may contribute to increased suicide risk and functional decline [17].

At the same time, cognitive approaches began to shed light on the mechanisms underlying these differences. Iqbal et al. approached diagnostic complexity from a cognitive perspective. Their research showed that patients with PPD recalled past experiences with a strong negative bias and did not have access to specific positive autobiographical memories. These memory patterns, while similar to those found in depressive disorders, seemed to reflect a vulnerability feature rather than a direct effect of psychosis or negative symptomatology. Such findings suggest that although cognitive features may resemble primary depression, in PPD, they arise from different mechanisms related to identity reconstruction and narrative processing after psychosis [19].

More recent studies have further emphasized the role of insight and psychological reintegration in this process. Moritz et al. approached diagnostic confusion from the perspective of insight and psychological reintegration. He proposed that paranoia may act as a temporary psychological buffer that protects individuals from confronting painful truths. The author proposed that paranoia may temporarily buffer self-awareness, underscoring the importance of phase and narrative context in differential diagnosis [26].

Overall, these studies emphasize that PPD diagnosis must consider the timing of onset, the presence of insight, the emotional content of symptoms, and their relationship to previous psychotic episodes. Although it is impossible to avoid overlap between negative symptoms, the effects of pharmacological treatment, and primary mood disorders, careful clinical assessment supported by targeted tools such as the Calgary Depression Scale remains essential to distinguish PPD as a clinically significant and independent condition. A historical but still useful framework, summarized by Kohler and Lallart, underscores timing (post-remission), exclusion of negative-symptom and iatrogenic states, and mood-specific assessment as anchors for differential diagnosis [18].

The differentiation of PPD from related affective and psychological constructs remains a significant clinical challenge. To clarify these distinctions, Table 1 provides a comparative overview of post-psychotic depression, secondary depression, demoralization, and post-psychotic trauma, highlighting key differences in timing, insight, affective content, narrative features, and treatment implications.

### 3.6. Treatment Approaches and Clinical Implications

Effective PPD treatment requires a profound understanding of both pharmacological and psychological interventions, as well as their timing in relation to the psychotic episode.

Jeczmien et al. addressed the pharmacological aspect of treatment, arguing that antidepressants can be effective in alleviating PPD symptoms in both the short- and long-term. However, they warned that responses to treatment are typically slower and less intense than in primary depression. Furthermore, they found that early withdrawal of antidepressants often led to a relapse of both depressive and psychotic symptoms, highlighting the need for long-term and closely monitored treatment strategies [15].

These observations were later integrated into a broader, phase-specific therapeutic framework by Kohler and Lallart. Their review emphasized the importance of combining pharmacological, psychological, and social interventions in phase-specific treatment of PPD [18].

The limitations of current pharmacological strategies were further illustrated by Buckley et al., who investigated the neurobiological links between antipsychotic treatment and symptoms of depression. The authors pointed out that some antipsychotic drugs, including risperidone and olanzapine, may exacerbate affective flattening or emotional blunting in some patients. Furthermore, he emphasized the role of hippocampal dysregulation as a potential biomarker for relapse of depression in schizophrenia, suggesting that treatment strategies should account for underlying neurotoxicity and neuronal sensitivity to dopaminergic blockade. These observations illustrate the pharmacological ambiguity of PPD. On the one hand, treatment with antipsychotic medications alone may contribute to emotional blunting and the emergence of depressive symptoms, making it difficult to distinguish treatment effects from negative symptoms and PPD, as Buckley et al. have already suggested [23].

On the other hand, attempts at pharmacological treatment of depressive symptoms have shown mixed results. Gregory and Mallikarjun demonstrated that antidepressants, particularly selective serotonin reuptake inhibitors, may be beneficial in some patients, although their overall efficacy remained less consistent than in cases of primary depressive disorders. This therapeutic uncertainty is reflected in the lack of clear guideline-based recommendations, which Dondé et al. identified as a persistent problem in the treatment of depressive symptoms in schizophrenia [33,34].

Subsequent studies suggest the possibility that some atypical antipsychotics may affect both the psychotic and affective dimensions of the disorder. In this context, Oguchi et al. described clinical improvement following a switch to lurasidone, while Carreras et al. noted a lower incidence of depressive complications in patients treated with the same drug following substance-induced psychoses. Taken together, these findings suggest that pharmacological factors may both contribute to the development of and alleviate depressive states following psychoses, further complicating their clinical interpretation [27,35].

Bressan et al. argued that strict ICD-10 time criteria may exclude patients from appropriate treatment and advocated a more flexible, symptom-evolution approach [16].

Meanwhile, psychological and cognitive interventions have been identified as protective factors. Moritz et al. contributed to this framework by introducing metacognitive training as a strategy to prevent affective breakdown after remission. Structural interventions that support cognitive reframing and rebuild self-esteem may serve as protective factors against PPD [26].

Despite these advances, there remains a gap between theory and clinical practice. Dondé et al. identified a persistent gap between diagnostic recognition and clinical application, noting that national psychiatric guidelines rarely include specific recommendations for the management of PPD. This lack of specificity continues to limit the implementation of evidence-based tools such as the Calgary Depression Scale [34].

In summary, treatment options for PPD remain limited and lack consistent scientific evidence to support them. Furthermore, there is no clearly defined standard of care. Table 2 provides a structured summary of the treatment strategies discussed in this review and their clinical implications.

## 4. Discussion

Psychological and cognitive studies emphasize the importance of self-esteem, humiliation, and autobiographical memory impairment in the development of PPD [11,12,19,20]. Patients with high levels of insight are more vulnerable to feelings of guilt and hopelessness after the disappearance of psychotic defense mechanisms. Social disintegration, loss of employment, and stigmatization further reinforce these depressive pathways. The overlap of these mechanisms explains the diagnostic complexity of PPD and its frequent misinterpretation as negative symptoms or the effects of pharmacological treatment.

The analyzed results also highlight the critical clinical consequences of this syndrome. Suicidal tendencies peak during remission, when insight and demoralization coexist [11,21,24,31]. This paradox, in which improved awareness increases psychological distress, remains one of the greatest challenges for clinicians. Therefore, timely diagnosis of PPD and active mood monitoring during recovery are essential to reduce risk and improve treatment outcomes.

From a therapeutic perspective, the literature suggests that both pharmacological and psychosocial approaches are needed. Traditional antidepressants may provide partial benefits but require long-term supervision to prevent relapse. Atypical antipsychotics with antidepressant properties, particularly lurasidone, show promise as dual-action agents targeting both psychotic and affective dimensions [27,35]. Equally important are psychological interventions aimed at rebuilding identity, narrative coherence, and self-esteem after psychosis.

Despite significant advances, research on PPD remains limited due to methodological inconsistencies. Many studies are based on small samples, lack standard diagnostic criteria, or do not include long-term follow-up. The lack of formal recognition of PPD in DSM-5 and its inconsistent representation in various diagnostic systems contribute to diagnostic uncertainty and clinical underdiagnosis. Future research should combine biological, cognitive, and social aspects, using validated tools such as the Calgary Depression Scale to ensure sample comparability [14,16,34].

Overall, a phase-specific approach combining pharmacological and psychosocial strategies is needed, with particular vigilance during remission, when the risk of affective disorders peaks [18,28].

## 5. Limitations

Several limitations of this review should be acknowledged. First, it was designed as a narrative synthesis rather than a systematic review, which precludes formal quality assessment and quantitative comparison of the studies included. Second, the literature search was limited primarily to the PubMed database, and relevant publications indexed elsewhere may not have been included. Third, narrative integration carries an inherent risk of selection and publication bias, particularly given the historical heterogeneity of the PPD literature. Fourth, the conceptual framework adopted in this review emphasizes insight-related and psychological mechanisms, which may result in insufficient consideration of alternative explanations, such as neuroprogressive or transdiagnostic models.

## 6. Conclusions

PPD should not be treated as a mere symptom accompanying schizophrenia, but rather as a distinct clinical phase requiring early identification, contextual understanding, and phase-specific interventions. Distinguishing PPD from negative symptoms, demoralization, and primary affective disorders remains a major diagnostic challenge. Tools such as the Calgary Depression Scale and more recent metacognitive models support more accurate classification.

Pharmacological options such as lurasidone appear promising for the treatment of both psychotic and affective symptoms, although their role must be interpreted alongside earlier evidence on antidepressant efficacy and relapse risk.

Future research should focus on longitudinal designs integrating neurobiological and psychological perspectives, with greater attention to patients’ subjective experiences during remission.

## Figures and Tables

**Table 1:** Conceptual and clinical distinctions between PPD, secondary depression and related constructs.

	Post-Psychotic Depression (PPD)	Secondary Depression	Demoralization	Post-Psychotic Trauma
Timing	After remission of psychosis	During or after psychosis	After losses, chronic stress	After psychosis or hospitalization
Phase specificity	Yes (remission phase)	No	No	Partial
Insight	High/increasing	Variable/often limited	Preserved	Usually preserved (reality testing intact)
Role of insight	Pathogenic (insight → distress)	Not central; may contribute but not required	Awareness of failure	Meaning-making of traumatic experience
Affective content	Shame, guilt, hopelessness, suicidality	Sadness, anhedonia, guilt	Hopelessness, resignation	Fear, shame, intrusive distress
Psychotic symptoms	Absent or residual	Often coexists (during active or residual psychosis)	Absent; may co-occur with residual symptoms	Absent
Narrative features	Identity loss, narrative fragmentation, overgeneral autobiographical memory	Negative but coherent	Meaning erosion	Trauma-centered narrative
Trajectory	Delayed onset; may remit with targeted care	Episodic or persistent	Fluctuating, context-dependent	Persistent or fluctuating; may become chronic if untreated
Suicide risk	High (early remission)	Moderate-high	Moderate-high (especially with avoidance/intrusions)	Moderate
Treatment implications	Antidepressants + phase-specific psychotherapy; consider atypical antipsychotics (e.g., lurasidone)	Antidepressants, antipsychotic adjustment (optional)	Psychosocial, meaning-focused interventions	Trauma-focused psychotherapy
Clinical priority	Active monitoring during remission	Mood stabilization	Contextual support	Trauma processing

**Table 2:** Treatment approaches in PPD based on reviewed studies.

Approach Category	Treatment methods	Key Findings	Clinical Implications
Pharmacological (Antidepressants)	SSRIs (citalopram, sertraline) [33]	Antidepressants may improve depressive symptoms, although results are variable [33].	Early discontinuation may be associated with relapse and requires careful monitoring [15].
Pharmacological (Atypical antipsychotics)	Lurasidone [27,35]	Lurasidone has been associated with improvement in depressive symptoms and reduced incidence of PPD [27,35].	May be considered in selected cases due to potential dual antipsychotic and antidepressant effects.
Psychological and Cognitive Interventions	Metacognitive training, cognitive approaches [26,30]	These interventions support recovery by addressing maladaptive beliefs and coping processes [26,30].	Useful in addressing insight-related distress and supporting adaptation after remission.
Integrated and Phase-Specific Frameworks	Phase-specific therapeutic approaches [18]	Multidimensional treatment models integrate pharmacological and psychosocial strategies [18].	Treatment should be tailored to illness phase and individual clinical context.
Clinical Management and Monitoring	Active monitoring and flexible assessment [11,21,31]	Early remission is associated with increased vulnerability to depressive symptoms [21,31].	Requires close monitoring and individualized diagnostic approach to distinguish PPD from negative symptoms [11].

## Data Availability

No new datasets were generated or analyzed during this study.
